# Perceptual chunking and its effect on memory in speech processing: ERP and behavioral evidence

**DOI:** 10.3389/fpsyg.2014.00220

**Published:** 2014-03-19

**Authors:** Annie C. Gilbert, Victor J. Boucher, Boutheina Jemel

**Affiliations:** ^1^Laboratoire de Sciences Phonétiques, Département de Linguistique et de Traduction, Université de Montréal, MontréalQC, Canada; ^2^Laboratoire de Recherche en Neurosciences et Électrophysiologie Cognitive, Hôpital Rivière-des-Prairies, MontréalQC, Canada; ^3^Centre de Recherche Fernand-Seguin, École d’Orthophonie et d’Audiologie, Université de Montréal, MontréalQC, Canada

**Keywords:** chunking, working memory, prosody, ERP, N400, P300

## Abstract

We examined how perceptual chunks of varying size in utterances can influence immediate memory of heard items (monosyllabic words). Using behavioral measures and event-related potentials (N400) we evaluated the *quality* of the memory trace for targets taken from perceived temporal groups (TGs) of three and four items. Variations in the amplitude of the N400 showed a better memory trace for items presented in TGs of three compared to those in groups of four. Analyses of behavioral responses along with P300 components also revealed effects of chunk position in the utterance. This is the first study to measure the online effects of perceptual chunks on the memory trace of spoken items. Taken together, the N400 and P300 responses demonstrate that the perceptual chunking of speech facilitates information buffering and a processing on a chunk-by-chunk basis.

## INTRODUCTION

The ability to interpret speech sounds inherently requires that rapidly changing sequences be kept in short-term memory (or “working memory,” Baddeley, 2010). However, since short-term memory is limited, one must assume that speech processing operates over some chunk of signal that fits this limited store (Kurby and Zacks, 2008; Ezzyat and Davachi, 2011; Farrell, 2012). On this idea, a number of authors argue that there is a basic perceptual or sensori-motor chunking that groups sequential stimuli (see Graybiel, 1998; Gobet et al., 2001; Terrace, 2001). This differs from the conventional notion of chunking suggested by Miller (1956), which involves a conceptual or semantic recoding of information. Miller’s chunking stands as a central concept of cognitive psychology. It is essentially defined as a strategy to enhance memory by grouping items in terms of varying semantic attributes, as in the classic example of letter sequences “*I, B, M, F, B, I, C, I, A, I, R, S*” being recalled in terms of the acronyms “*I.B.M., F.B.I., C.I.A., I.R.S.*” Perceptual chunking, on the other hand, is described by Gobet and Terrace as an automatic perceptual process that is domain-general and that creates groups in sequential stimuli. Such grouping is commonly observed in sequence learning tasks. For instance, in learning and producing novel lists of digits or nonsense syllables, temporal groups (TGs) arise spontaneously. These perceptual chunks or groups are generally marked by characteristic delays or a lengthening of inter-response times at the ends of groups (Terrace, 2001). It is known that perceptual chunks do not tend to exceed four items, which conforms to the capacity limits of short-term serial memory (for an extensive discussion of chunk limits in sequence recall, see Cowan, 2000). Moreover, numerous studies have shown that grouping items in three or four benefits sequence memory (Wickelgren, 1964; Broadbent and Broadbent, 1973; Frankish, 1989, 1995; Hitch et al., 1996; Cowan, 2000; Reeves et al., 2000; Terrace, 2001; Mayberry et al., 2002; Chen and Cowan, 2005; Boucher, 2006). What is less recognized, though, is that temporal grouping marked by length changes also operates in speech production and perception. For instance, a number of studies attests to the production of variably termed “accentual” groups in natural speech that do not tend to exceed four syllables on average (e.g., Dauer, 1983; Martin, 1999; Boucher, 2006), while other studies suggest that listeners detect such groups in speech (Nooteboom, 1997; Christophe et al., 2003, 2004; Boucher, 2006; Gilbert et al., 2010; Gilbert, 2012).

Aside from behavioral observations, a recent study by Gilbert et al. (2010), Gilbert (2012) using event-related potentials (ERPs) has provided more direct evidence of the perceptual chunking of heard speech in terms of TGs. In particular, the study showed that a neural component called *closure positive shift* (CPS) is evoked by lengthening marks of groups -independently of the intonation patterns or the syntactic-semantic content of utterances. In other words, the evoked CPS response revealed that listeners chunk speech in terms of TGs similar to those that appear spontaneously in sequence learning across behaviors (Terrace, 2001). As such, the CPS component is characterized by an incremental negativity over midline central sites followed by a rapid positive deflection. This rising negativity across items in a group is suggestive of a buffering of information that ends with a positive shift corresponding to a group-final mark. The CPS has previously been associated with intonation and semantic-syntactic units (cf. Steinhauer et al., 1999; Steinhauer and Friederici, 2001; Kerkhofs et al., 2009; Bögels et al., 2010; Dilley et al., 2010; Itzhak et al., 2010; Brown et al., 2011; Pauker et al., 2011). Though the new evidence of Gilbert clarifies that CPS can specifically reflect a perceptual chunking of speech in TGs, it remains unclear whether such chunks could be linked to processes of working memory. If indeed this is the case, then one might expect that TGs of differing size would influence listeners’ immediate memory of speech.

In assessing this hypothesis, we focus in the present report on how perceived groups in utterances variably affect the memory trace of heard items. In our test, we use an adapted Sternberg task where listeners hear an utterance followed by a target word and are asked to indicate, as fast as possible, whether or not the target was part of the utterance.

First, one should keep in mind that the Sternberg task is a memory scanning paradigm. Results on this task generally show that increasing the number of verbal items in a presented set leads to an increase in response times to given targets by about 30 to 40 ms per item (Sternberg, 1966). This linear relation between the number of items in a set and response times suggests that participants are scanning the entire content of their working memory before responding. In terms of the Sternberg paradigm, such scanning does not imply activation of every stored item but does imply some process of comparison between a target and a memory set. Only when a match is established is there *activation* or access to an element in the set leading to a response. Furthermore, the ERP literature on memory scanning has generally focused on two ERP components: the P300 and N400 components (see Wolach and Pratt, 2001; Kutas and Federmeier, 2011 for reviews). Whereas the N400 may mostly relate to the activation of items, research has shown that the P300 is associated with stimulus comparison and classification during the scanning process (Kutas et al., 1977; Magliero et al., 1984). Note that, through the years, the P300 has been known to reflect the sum of different components associated to different mechanism rather than a single response (including the P3a, P3b, and Novelty P3, see Polich, 2007 for a review). Here, we refer to the positive response peaking around 300 ms from the stimulus onset using the generic label “P300” to avoid taking a stand on the specific underlying processes involved, which fall beyond the scope of the present report (Steiner et al., 2013).

A number of studies have reported that the amplitude of P300 evoked by a target decreases as the size of the memory set increases (see, e.g., Marsh, 1975; Gomer et al., 1976; Karrer et al., 1980; Starr and Barrett, 1987; Pratt et al., 1989; Pelosi et al., 1995; Houlihan et al., 1998; Wolach and Pratt, 2001). P300 latencies have also been related to stimulus classification times (Kutas et al., 1977; Duncan-Johnson and Donchin, 1982; McCarthy and Donchin, 1983; Magliero et al., 1984), though there is some disagreement on this point. Several studies show significant increases in P300 latency for a target when sets increase in size (Marsh, 1975; Gomer et al., 1976; Adam and Collins, 1978; Ford et al., 1979, 1982; Pfefferbaum et al., 1980; Kramer et al., 1986; Starr and Barrett, 1987; Pratt et al., 1989) but some show no significant change beyond sets of two or three items (Pfefferbaum et al., 1980; Pelosi et al., 1992, 1995). In the present report, the P300 component is specifically used to evaluate effects of perceptual chunking on the scanning of working memory.

As for the N400, though this component was first thought to reflect the degree of semantic integration of an item to a preceding sentence (Kutas and Hillyard, 1980), it is now associated with effort relating to the activation (or accessing) of an element in a presented context (for a review, see Kutas and Federmeier, 2011). More specifically, the amplitude of the N400 varies inversely with the degree of pre-activation of an item: the more restricted a context is, the more an item gets pre-activated and the smaller the N400 effect (Kutas and Federmeier, 2011; Amoruso et al., 2013; Strauß et al., 2013). This means that the relative amplitude of the N400 can index the quality of the memory trace of an item. An item with a strong memory trace would be easier to recall and generate a smaller-amplitude N400 than an item with a poorer memory trace. Hence, the N400 may serve to evaluate effects of perceptual chunks of varying size on the quality of the memory trace of heard items.

In view of the general nature of chunking and the above evidence, we hypothesize that listeners perceptually chunk utterances in TGs and that this process links to immediate memory of heard speech. To demonstrate this link, we vary the size of TGs from three to four items. Groupings of three and four are known to affect memory differently; with groups of three having optimal benefits for sequence recall (Cowan, 2000; Boucher, 2006; Mathy and Feldman, 2012). In using a Sternberg task to study these effects, we predict that the accuracy of target recognition, response times, and N400 amplitudes will vary with the size of TGs. Furthermore, as we noted earlier, response times in a typical Sternberg task vary with the number of items in a set and item position is generally expected to have little effect. This is generally interpreted as suggesting that subjects are scanning the entire content of their working memory before responding. However, in our task, the presented stimuli are utterances containing TGs. In these contexts, then, items may be perceived in groups so that a scanning may operate by consecutive chunks. In other words, a match between a target and an item of the first chunk in working memory may lead to an earlier activation compared to a target in a following chunk. If this is the case, then response times to targets may vary in terms of whether they occur in the first or second perceptual group in an utterance, and this may be reflected at the level of P300 characteristics. Taken together, a validation of the above hypotheses provides a way of determining how perceptual chunking can influence working memory in speech processing.

## MATERIALS AND METHODS

### PARTICIPANTS

The participants were 16 native speakers of French, aged from 19 to 42 years (mean age = 25.6 years), who presented normal hearing levels following a standard audiometric screening. All were dominant right-handers (Oldfield, 1971), with no history of substance abuse (other than tobacco smoking), and no history of neurological or psychiatric disorder. All showed normal memory performances on the digit-span test of the WAIS (Wechsler, 1997; overall, average normalized score: 10.16, std dev.: 2.4). Written consent was obtained from every participant, and the present research protocol was approved by the ethics committee of the *Hôpital Rivière-des-Prairies* (Montréal, QC, Canada).

### STIMULI

**FIGURE 1:**
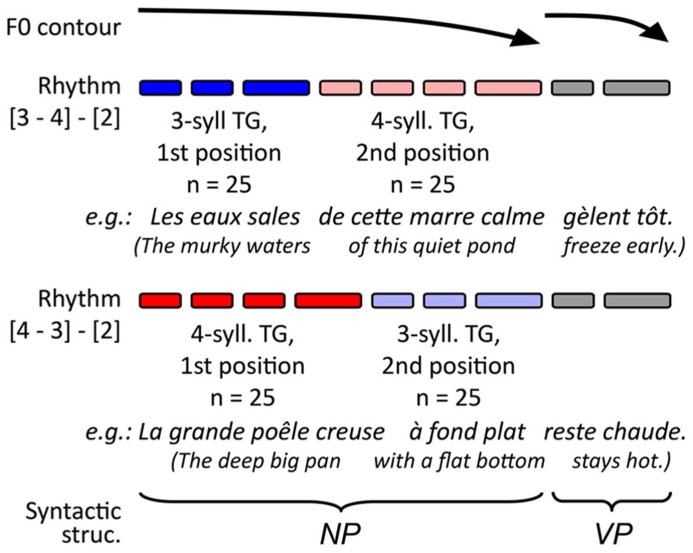
**Schematic representation of intended prosodic structure of the stimuli and the origin of the targets**.

The experimental stimuli for the present study consisted of 100 pairs of French utterances and target lexemes. The target lexemes were all monosyllabic nouns matched to the utterance context. Only nouns were used as targets to avoid any confounding effect of lexical class on the retrieval processes (Halgren et al., 2002). The utterances, on the other hand, were made up of monosyllabic lexemes and functors in a similar sentence structure. This structure allowed a speaker to produce the contexts with specific prosodic patterns and TGs, as summarized in **Figure 1**. A pacing technique (described below) served to control our experimental variables. These variables involved changing *TG length* (groups of three and four syllables, see blue and red lines in **Figure 1**) and *TG position* (first and second group in the utterance, see dark and light shades respectively). The target lexemes were distributed in equal numbers across conditions so that 25 targets were placed in each combination of TG length and TG position (giving four different sets of 25 stimuli). As for the potential effects of syntax, the grammatical function of the TGs was maintained across utterances: the first TG was always a subject NP, and the second a complement to the subject NP (the third TG, which was VP, is not relevant in the present study; the full lists of utterance contexts can be obtained upon request via email to the first author). In all TGs, the target noun was a pre-final element, which served to avoid potential recency effects. Moreover, across conditions, the target nouns used had comparable frequency indices in French [*F*(3,94) < 0.517; MSE > 11,710; *p > *0.672), Desrochers, 2006].

In order to create natural sounding contexts, the recording of the target nouns and utterances was done by a male native speaker of French using a pacing technique. By this technique, the speaker follows heard series of rhythmic tones (a metronome-like pacer) in producing utterances with the desired prosody. (For further details of the pacing technique, we refer to Gilbert, 2012). The speaker’s productions were recorded using a 16-bit external soundcard (*Fast-track Ultra*, M-Audio) at a sampling rate of 44.1 kHz and stored in separate wav files. The target lexemes were recorded separately (and not spliced from the original utterance to avoid coarticulation effects) and this provided natural sounding prosody that was appropriate for isolated lexemes in French (Fant et al., 1991). The target lexemes lasted 447.32 ms on average (minimum 425 ms, maximum 477 ms) and bore a neutral tone.

Finally, to tag accurately the onset of target words in the electroencephalography, we relied on the perceptual-center (*P-center*) of the target lexemes. The *P-center* constitutes the point in time where a syllable is perceived (Marcus, 1981). Such measures are required to minimize the latency jitter when averaging the ERPs and to allow for precise calculation of intervals between the target and the participant’s response. In particular, we made sure that constant intervals were maintained between the *P-center* of the target lexeme and both the onset and offset of its sound file. In the present case, the *P-center* of the monosyllabic target was always 200 ms from the beginning and 300 ms from the end of the file. Audio file editing and amplitude normalization was performed using *GoldWave* (GoldWave Inc., v5.58).

### PROCEDURES

**FIGURE 2:**
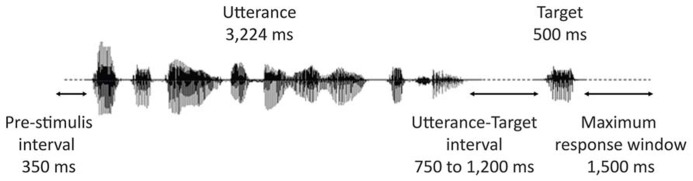
**Time-course of a trial**.

**Figure 2** represents the time-course of a typical trial. All contexts were presented using insert earphones (*Eartone* *3A, *EAR Auditory Systems). The sounds were delivered at a constant intensity, which was calibrated with a sonometer to obtain peak levels of 68 dBA (i.e., a conversational-level sound volume) at the inserts. Participants were instructed to listen to the stimuli while maintaining their gaze at a fixation point on a blank screen. They were also instructed to keep index fingers of both hands on two buttons of a response box. Their task was to indicate, as fast as possible by a key press, whether or not the prompt (the target lexeme) was part of the preceding utterance or not. Half the participants answered affirmatively by pressing the right button with their right hand, half by pressing the left button with their left hand. Sound files were played back via *E-prime 1.0* (Psychology Software Tools) in random blocks divided by rest pauses. Each block contained on average 40 stimuli (eight targets and 32 stimuli belonging to different experimental conditions not reported here) delivered in random order with the restriction that no consecutive stimuli presented the same prosodic pattern. The interval between the end of an utterance and the beginning of the audio file of the target lexeme varied from 750 to 1,200 ms in steps of 50 ms (11 different intervals). Presentation of successive trials was initiated by the participants’ response or automatically after 1,500 ms if no response was given.

### EEG RECORDING AND ERP ANALYSIS

The electroencephalography (EEG) data were recorded through shielded electrodes embedded in an elastic cap (*Easy Cap*) according to the enhanced 10–20 system (Sharbrough et al., 1991). Two bipolar electrodes placed above and below the dominant eye [vertical electrooculography (EOG)] and at the outer canthus of each eye (horizontal EOG) served to record eye movements and blinks. A left mastoid electrode was used as an online reference for all scalp electrodes, and AFz served as the ground. The EEG including EOG signals were recorded continuously with a band-pass from DC to 100 Hz at a sampling rate of 512 Hz, and stored with trigger codes. The EEG signal was average-referenced offline and filtered using a 10 Hz low-pass Butterworth of the sixth order with zero-lag, yielding a -36 dB/octave roll-off. Only trials corresponding to correct responses were kept and submitted to the further procedures. EEG segments with eye-blinks and other artifacts were automatically rejected if (i) the standard deviation of the EOG channels within a 200 ms sliding window exceeds 40 μV and if (ii) the standard deviation of any scalp electrode exceeds 20 μV. Eye blinks were then detected and corrected by subtracting from the EEG the PCA-transformed EOG components for each electrode, weighed according to VEOG propagation factors (computed using linear regression). Segments with other artifacts were rejected.

The obtained artifact-free EEG segments, which were time-locked to the onset of a target sound file, were then averaged for each group length and group position, from 100 ms before to 700 ms after the onset of a target sound file. The ERPs were also pooled together according to the TG length irrespective of TG position and vice-versa. This allowed for the calculation of difference waves isolating one factor from the other. Time-sequence topographies of the differential waveforms were used to identify the regions of interest (ROIs). Specifically, we selected the ROIs based upon a visual inspection of the data, taking into account both the individual averages and group average. For the N400 effects, our visual inspection focused on the size of the ERP amplitude difference in the traditional N400 time-window. As for the P300, ROIs were selected based on the sites that showed the largest positive deflections within the P300 time-window. This allowed the selection of the sites showing the most conspicuous N400 and P300 effects. Each participant’s ERP was re-averaged according to the ROIs and these were used to obtain quantitative measures of peak latency and amplitude (maximal and averaged within a time-window). The N400 was identified as the relative negativity peaking between 300 and 500 ms post-stimulus, (Kutas et al., 1977; Kutas and Federmeier, 2011) over central sites (including FCz, Cz, and C2) whereas the P300 was defined as the positive maximum peaking around 300 ms, immediately before the negative deflexion associated to the N400, over central-parietal sites (including Cz, C1, C2, CPz, CP1, and CP2).

All statistical analyses were computed using 2X2 analysis of variances (ANOVAS) with TG length (three vs. four) and position in the utterance (first vs. second) as implemented in SPSS (version 17.0).

## RESULTS

### BEHAVIORAL DATA

The recognition task was easy and correct recognition was high, with scores varying from 92 to 96% correct (see **Table 1**). A 2X2 ANOVA showed no main effects of TG length or position [*F*(1,15) < 1.561; MSE = 0.004; *p > *0.23; η^2^ < 0.095] and no significant interaction [*F*(1,15) = 2.529; MSE = 0.002; *p = *0.133; η^2^ = 0.144]. As for the reaction times (RTs) for correct responses, a significant main effect was found for the position of the TGs [*F*(1,15) = 9.638; MSE = 1,149; *p *= 0.007; η^2^ = 0.391], target words pertaining to the first group triggering shorter RT than those pertaining to the second. However, there were no significant effects of TG length [*F*(1,15) = 1.047; MSE = 2,224 *p *=**0.322; η^2^ = 0.065], and no significant interaction [*F*(1,15) = 2.142; MSE = 2,617; *p *= 0.164; η^2^ = 0.125]. Overall, the analyses showed no marked influence of the size of chunks on subjects’ behavioral responses, only an effect of TG position in the utterance.

**Table 1:** Behavioral results: means and standard deviations of correct recognition scores and RT as a function of TG length and position.

	TG length	
	Three syll.		Four syll.	
TG position	Accuracy	RT (ms)	Accuracy	RT (ms)
	*SD*	*SD*	*SD*	*SD*
First	92.2	708.7	95.8	677.92
	*0.045*	*183.5*	*0.052*	*147.06*
Second	94.9	716.29	95.3	722.94
	*0.076*	*166.78*	*0.049*	*170.92*

### ERP DATA

The analyses of the continuous EEG focused on two different time-windows previously associated with the P300 and N400 ERPs (described in section 2.4 EEG recording and ERP analysis). Here we present the results obtained for both components separately.

#### P300

**FIGURE 3:**
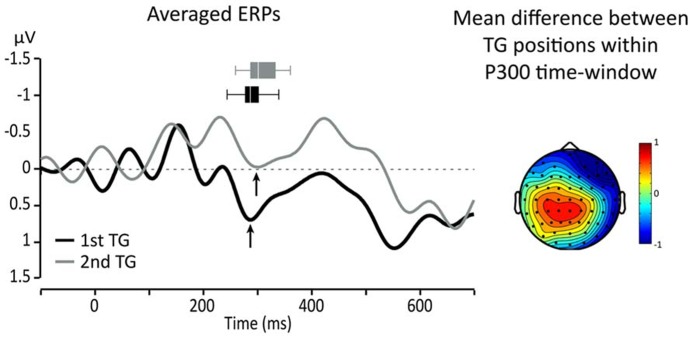
**Left: averaged responses for centro-parietal ROI: black line = first TG, gray line = second TG.** Arrows indicate the peak of the P300 and horizontal box plots represent peak latency dispersion among participants. Right: topography of the average difference between ERPs for targets from TGs in first and second positions in the utterance.

Since we expected that the P300 would be affected by the position of TGs, topographical maps of the differences between targets from the two TG positions (first and second in the utterance) were used to identify ROI and relevant time-windows. A visual inspection showed that the largest difference was in a central-parietal region which includes Cz, C1, C2, CPz, CP1, and CP2. **Figure 3** presents topographical ERP difference for TG positions in the time-window of the P300 and the ERP waveforms re-averaged according to the identified ROI (black line = TG in first position, gray line = TG in second position). One can see that the difference between the conditions extends outside the range of the P300. In fact, TGs in second position present, overall, a smaller positivity between 200 and 600 ms than TGs in the first position (black line).

**FIGURE 4:**
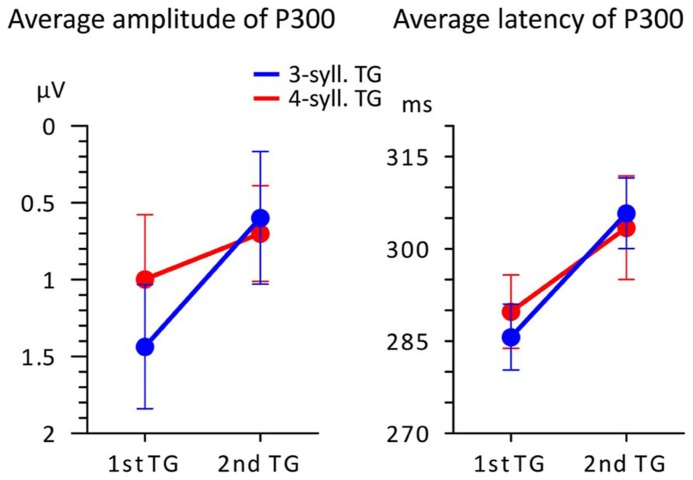
**Average amplitude and latency (*n* = 16) of the P300 peak plotted according to TG position (first vs. second) and TG length (blue = 3-syll. vs. red = 4-syll.).** Vertical bars represent standard errors.

To evaluate the effect of the experimental conditions on these differences, we focused on the P300 component and measured the average amplitude (per participant) over five consecutive 25 ms time-windows starting at 200 ms post-onset. For all of these measures, a 2X2 ANOVA comparing TG length (three- vs. four-syll.) and position (first vs. second) showed no significant main effects [*F*(1,15) < 2.879; MSE > 1.122; *p > *0.1.; η^2^ < 0.161] or interactions [*F*(1,15) < 3.989; MSE > 1.064; *p *> 0.06; η^2^ < 0.21]. Peak amplitude and latency measures were also taken on the P300 for each participant. The P300 was identified on the average waveform as the positive peak immediately preceding the negative deflection of the N400 as indicated by the arrows in **Figure 3**. The results of these latter measures are displayed in **Figure 4**.

One can see in the left panel of this figure that targets evoke greater P300 amplitudes when they are heard in the first position compared to when they are heard in the second position of the utterance. However, a 2X2 ANOVA showed that main effects and interactions were not significant [*F*(1,15) < 3.374; MSE > 0.907; *p *> 0.08; η^2^ < 0.206]. Moreover, what appears as an effect of TG length in the first position was found to be non-significant in terms of a repeated measure *t*-test [*T*(1,15) = 1.249; *p *= 0.231.; η^2^ = 0.094]. This likely owes to the small changes in “set sizes” of TGs and the simplicity of the task (see Discussion and Conclusion for an explanation of the seeming discrepancy between the changing neural responses in **Figures 3** and **4** and the statistical results). On the other hand, P300 peak latencies (seen in the right panel of **Figure 4**) present marked differences with respect to position. A 2X2 ANOVA confirmed that only TG position had a significant impact on P300 latency [*F*(1,15) = 10.865; MSE = 0.000; *p *=**0.006; η^2^ = 0.455] with targets from first TGs triggering earlier P300s than targets from the second TGs. The statistical analyses yielded no main effect of TG length and no interactions [*F*(1,15) < 0.098; MSE = 0.001; *p* > 0.759; η^2^ < 0.008].

#### N400

**FIGURE 5:**
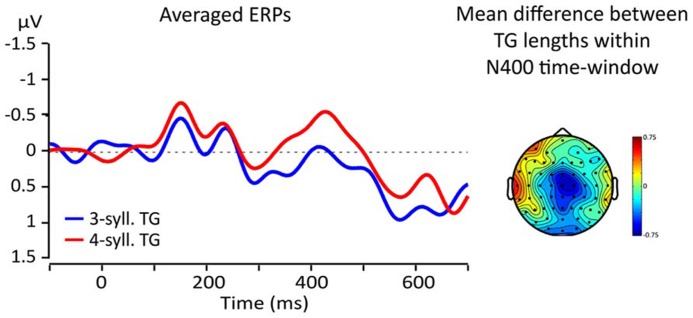
**Left: averaged responses for the fronto-central ROI; blue lines = 3-syll TG, red lines = 4-syll TG.** Right: topography of the average differences between ERPs for targets from TGs of different lengths (four- minus three-syll.) in the 350–450 ms time-window.

Visual inspections of the topographical difference between TGs of three and four syllables revealed that the greatest difference was found in a region comprising electrodes FCz, Cz, and C2 (ROI) and at latencies within the usual time-window of the N400 effect (from 300 to 500 ms, Kutas et al., 1977; Kutas and Federmeier, 2011). **Figure 5** shows the topographical distribution of the mean amplitude difference between TG lengths (three vs. four) within a 100 ms time-window from 350 to 450 ms and the ERPs re-averaged according to the ROI. Line color represents TG length (three-syll. in blue, four-syll. in red). Note that the four-syllable TGs elicit a larger N400 than the three-syllable TGs.

**FIGURE 6:**
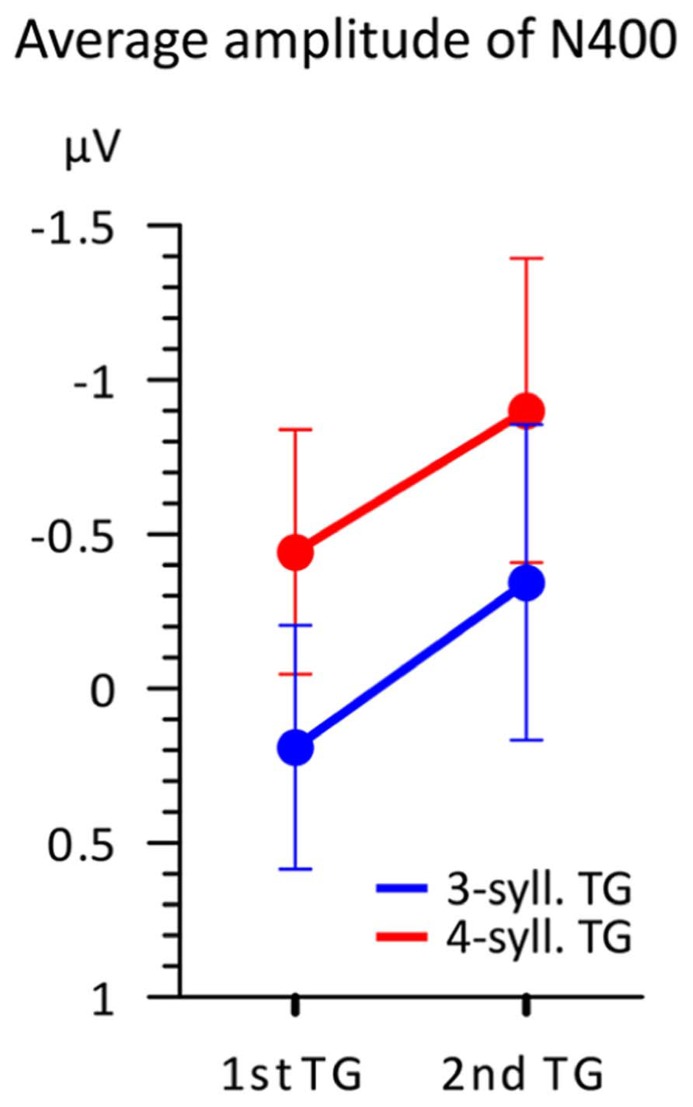
** Average amplitude (*n* = 16) in the 350–450 ms time-window according to both TG position (first vs. second) and TG length (blue = 3-syll. vs. red = 4-syll.).** Vertical bars represent standard errors.

This N400 amplitude difference is further illustrated in **Figure 6** representing the mean amplitudes in the 100 ms time-window according to both TG length and position. This figure illustrates two main findings. First, TGs of three syllables display a smaller negativity than TGs of four syllables when controlling for position. Second, the N400 mean amplitude is greater overall for TGs in the second position than that from first position. Therefore, it seems that two factors influence the amplitude of the N400. However, a 2X2 ANOVA comparing TG length (three- vs. four-syll) and position (first vs. second in utterance) showed a significant effect only for TG length [*F*(1,15) = 8.506; MSE = 0.666; *p *= 0.011; η^2^ = 0.362]. Main effects of TG position and interactions were not significant: [*F*(1,15) < 1.152; MSE > 0.587; *p > *0.3; η^2^ < 0.071].

## DISCUSSION AND CONCLUSION

In the above task, listeners heard utterances followed by a word item and were asked to rapidly indicate whether or not the target word was part of the heard utterances. Targets within the contexts were placed in perceptual chunks of different size (three or four items) and these chunks appeared in different positions (first or second) in an utterance. The results show that TG size had a significant impact on the quality of the memory trace as reflected in the amplitude of the N400. This effect was not reflected in behavioral responses of recognition accuracy, largely because the task of recalling items in heard utterances can be quite easy, even more so when items are in groups that match the span of working memory (Cowan, 2000). Hence, the task was hardly demanding and this led to high recognition scores with only slight fluctuations across conditions. Nonetheless, the size the TGs had, as predicted, a significant effect on the amplitude of the N400 component validating the view that perceptual chunks in utterances link to immediate memory of speech. Additional effects were studied in terms of the memory scanning task that confirmed that speech in working memory is processed on a chunk-by-chunk basis.

To clarify this last point, it should be recalled that the Sternberg task provides a means of assessing how items are processed in short-term working memory. The above results show significant effects of the position of TGs on RTs. Thus, even though the utterances were of the same length (nine monosyllabic words), the recognition times were shorter when target items were heard in the first group than when they were heard in the second group. To account for such differences, one has to assume that the content of working memory was scanned in terms of ordered chunks and stopped at a point when there was a match between the target and an item in a chunk. Hence, it appears that utterance-related information is stored in serially-ordered chunks and that the scanning of this information in working memory proceeds on a chunk-by-chunk basis. Observations of the P300 also appear to support this interpretation, though this component could also be influenced by the location of subject forms in the presented contexts.

The P300 is generally associated with memory scanning processes, and its latency is believed by some to be linked to scanning durations (e.g., Pelosi et al., 1995). In the above observations, P300 latencies show effects of TG position that conform to duration differences in response times: essentially, TGs heard first in the utterances led to shorter response times and earlier P300s compared to later-occurring TGs. It may be useful to mention that these results agree with reports of a relationship between P300 latencies and “set size,” where sets in the present experiment are given by TGs (for a review, see Pelosi et al., 1995). That is, the difference with respect to previous work is that the Sternberg task in the present study includes grouped stimuli. As for P300 amplitude, studies have reported decreases in P300 amplitudes for increases in set sizes (for a review, see Wolach and Pratt, 2001). We also observed such patterns of decreasing P300 amplitude, as seen in **Figures 3** and **4**, though the differences did not reach statistical significance. Yet the results conform to general patterns found across studies: targets heard in TGs that are presented early in a context tend to be associated with larger-amplitude P300s compared to targets heard in later TGs. This seems to be even more marked for targets from short TGs compared to long TGs. The non-significance of the differences likely owes to the relative simplicity of the task or the small difference between our “set sizes” of three and four syllables. Nonetheless, the P300 responses strongly suggest a scanning of working memory on the basis of ordered groups or chunks. It may be noted that this interpretation stands in contrast to a number of behavioral studies involving the recall of visually presented letters or digits, which suggest that position effects on RTs may not reflect a mechanism of serial scanning (e.g., Townsend and Ashby, 1983; McElree and Dosher, 1989). On the other hand, recall of unstructured lists of letters or digits may not extrapolate to the processing of prosodically structured speech where the perception of chunks and memory of serial order is essential to the interpretation of utterances.

Overall, the above results, combined with the earlier findings of Gilbert et al. (2010), Gilbert (2012) offer a perspective on how heard speech is chunked and stored online. Basically, the findings suggest that listeners perceive speech in TGs, and these perceptual chunks are stored in working memory in a serial order. That this perceptual chunking of speech in TGs inherently links to memory processes is demonstrated by the effects of group size on the N400 amplitude. In weighing these findings, it is useful to remark that, despite the large body of research on chunking (for a review, see Cowan, 2000), there is a paucity of work on the role of perceptual chunking in speech processing. This can be explained by the fact that research on chunking is largely based on experiments involving recall whereas perceptual chunking requires a method that captures online responses to incoming stimuli (Terrace, 2001). In this context, the above research using ERPs provides a novel demonstration of how perceptual chunking influences immediate memory of heard speech. It should also be emphasized that the evidence indicating that listeners perceive speech in groups should not be equated with the idea that listeners are detecting prosodic phrases that map syntactic forms (e.g., Frazier et al., 2006). In fact, groups created by delays or a lengthening of elements generally emerge in learning and producing sequences in numerous behaviors both verbal and non-verbal (for examples, see Graybiel, 1998; Terrace, 2001). Hence, these TGs or chunks that arise spontaneously do not “map” syntactic-semantic structure. Instead, they reflect a domain-general sensori-motor chunking that links to working memory (Graybiel, 1998). This presents another perspective on the role of perceptual chunking in language processing that could extend to language learning. In this area, Beckner and Bybee (2009) have expressed the need to explain the emergence of multi-word chunks in language learning by some prelinguistic, domain-general process. The above findings may indicate that perceptual chunking can constitute one of those domain-general processes.

## Conflict of Interest Statement

The authors declare that the research was conducted in the absence of any commercial or financial relationships that could be construed as a potential conflict of interest.
